# Interplay of Oxidative Stress, Inflammation, and Autophagy in RAW 264.7 Murine Macrophage Cell Line Challenged with Si/SiO_2_ Quantum Dots

**DOI:** 10.3390/ma16145083

**Published:** 2023-07-19

**Authors:** Loredana Stanca, Ovidiu Ionut Geicu, Andreea Iren Serban, Anca Dinischiotu

**Affiliations:** 1 Preclinical Sciences Department, Faculty of Veterinary Medicine, University of Agronomical Sciences and Veterinary Medicine Bucharest, 105 Splaiul Independentei, 050097 Bucharest, Romania; loredana.stanca@fmvb.usamv.ro (L.S.); yo3hfh@yahoo.com (O.I.G.); 2 Department of Biochemistry and Molecular Biology, Faculty of Biology, University of Bucharest, 91–95 Splaiul Independentei, 050095 Bucharest, Romania

**Keywords:** Si/SiO_2_ QDs, RAW 264.7, oxidative stress, antioxidative enzymes, cell membrane damage, autophagy, inflammatory response, cytokines, heat shock proteins, immunotoxicity

## Abstract

Quantum dots (QDs) with photostable fluorescence are recommended for imaging applications; however, their effect on living cells is incompletely understood. We aimed to elucidate the RAW 264.7 murine macrophage cell line’s response to the Si/SiO_2_ QDs challenge. Cells were exposed to 5 and 15 μg/mL Si/SiO_2_ QDs for 6 h, 12 h, and 24 h. Cell metabolic activity and viability were assessed by MTT, live/dead, and dye-exclusion assays. Oxidative stress and membrane integrity were assessed by anion superoxide, malondialdehyde, and lactate dehydrogenase activity evaluations. Antioxidative enzyme activities were analyzed by kinetic spectrophotometric methods. Cytokines were analyzed with an antibody-based magnetic bead assay, PGE2 was assessed by ELISA, and Nrf-2, Bcl-2, Beclin 1, and the HSPs were analyzed by western blot. Autophagy levels were highlighted by fluorescence microscopy. The average IC50 dose for 6, 12, and 24 h was 16.1 ± 0.7 μg/mL. Although glutathione S-transferase and catalase were still upregulated after 24 h, superoxide dismutase was inhibited, which together allowed the gradual increase of malondialdehyde, anion superoxide, nitric oxide, and the loss of membrane integrity. G-CSF, IL-6, TNF-α, MIP-1β, MCP-1, Nrf-2, PGE2, and RANTES levels, as well as autophagy processes, were increased at all time intervals, as opposed to caspase 1 activity, COX-2, HSP60, and HSP70, which were only upregulated at the 6-h exposure interval. These results underscore that Si/SiO_2_ QDs possess significant immunotoxic effects on the RAW 264.7 macrophage cell line and stress the importance of developing effective strategies to mitigate their adverse impact.

## 1. Introduction

Image analysis is a crucial aspect of biological and medical research, with potential applications in the diagnosis, treatment, and monitoring of chronic diseases. Due to limitations of optical imaging such as the limited penetration depth of light in living tissues and the limited brightness and stability of conventional fluorophores [1], this type of imaging has not been as well developed for the medical or research fields as other imaging techniques, such as magnetic resonance spectroscopy, optical bioluminescence, ultrasound, and others [2,3]. 

Quantum dots (QDs) are resistant to photobleaching and emit photostable fluorescence; of particular interest are those emitting in the near IR region of the spectrum, where tissue autofluorescence is reduced and excitation light penetration is increased [4]. The emerging field of QDs use in biomedical imaging has shown promising results; however, their translation into clinical applications in humans is still in the early stages, in part due to partially explored safety ramifications [5]. Most studies involving QDs have been conducted in preclinical settings using in vitro experiments or animal models, and QDs use as therapeutic agents, drug carriers, or in medical imaging is still in the early stages of investigation [6,7,8,9,10]. Efforts to reduce nanoparticle toxicity due to their composition have included encapsulation in silicon shells, which has the added benefit of chemical flexibility and also makes the surface suitable for functionalization. Silicon is also a suitable candidate for dye doping, enabling the incorporation of dyes into the silica structure [5].

Due to their size, nanoparticles (<100 nm) and QDs (1.5–10 nm) can interact with biological structures such as cell membranes and organelles and proteins like cell receptors and enzymes. The physical presence of these nanoscale materials was shown to cause functional alterations of biological structures, even in the case of those made of inherently nontoxic materials [11,12,13]. In addition, reducing the size to the nanolevel has the effect of a dramatic increase in the surface-to-volume ratio, so that, percentage-wise, more constituent atoms of the nanoparticle are located on its surface, increasing the intrinsic toxic potential [14]. For this reason, nanoparticles are generally more toxic than larger particles with the same composition. 

Nanoparticles can invoke starkly different responses from specific cell types [15,16,17,18], and silica particle toxicity for macrophages is not a new phenomenon [19]. Research has shown that mononuclear phagocytic cells play a critical role in producing tissue damage associated with silicon nanoparticle exposure [20]. This selective toxicity of silica particles with a simple composition and low reactivity is intriguing. Therefore, understanding the mechanism of silica-induced macrophage death is important for developing strategies to mitigate the adverse effects of these particles. The working hypothesis for this study is that exposure to Si/SiO_2_ (QDs) triggers a cascade of cellular responses in RAW 264.7 macrophages involving oxidative stress markers, antioxidant enzymes, heat shock proteins, autophagy, and cell death pathways, potentially leading to immunotoxicity.

The RAW 264.7 murine macrophage cell line is extensively utilized due to its ability to faithfully replicate inflammatory reactions, thereby providing valuable insights into the immunomodulatory properties of tested substances [21]. Although more recent insights have brought into question the inherent differences between the immune responses of mouse and human cells [22], researchers have successfully employed RAW 264.7 cells as a convenient model to study macrophage biology and certain immune responses [23,24,25,26,27].

This study aims to elucidate the interplay between these cellular processes and the role of specific proteins in modulating inflammation and cell survival, ultimately contributing to a better understanding of macrophage sensitivity and the immunotoxic effects of Si/SiO_2_ QDs.

## 2. Materials and Methods

In this section, we provide a comprehensive overview of the methods employed in this study. Table 1 summarizes the assays utilized to investigate various aspects of cellular responses and function in order to provide a clear and organized outline of the experimental approaches employed throughout the study. 

### 2.1. Quantum Dots

The Si/SiO_2_ QDs utilized in this research were generated through pulsed laser ablation performed at the Laser Department of the National Institute of Lasers, Plasma, and Radiation Physics in Bucharest-Măgurele. The approach used to synthesize these QDs has been documented in previous publications [28,29,30]. The QDs used in this study were thoroughly described in previous papers published by our group [18,31]. Briefly, XRD diffraction peaks indicated the presence of crystalline silicon in the core of the QDs. Further examination using selected area electron diffraction imaging revealed that the QDs had a crystalline Si core surrounded by a 1–2 nm amorphous layer. TEM analysis showed that the QDs had a spherical morphology and diameters between 6 and 8 nm and tended to form irregularly shaped, closely packed nanoparticle aggregates that were disrupted and dispersed by sonication. The QD suspensions demonstrated visible red-orange fluorescent emission under UV excitation [17,28]

### 2.2. Cell Culture

The RAW 264.7 (subclone TIB-71) cell line (ATCC, Manassas, VA, USA) is derived from a murine Abelson leukemia virus-induced tumor and exhibits a monocyte/macrophage morphology. Cells were initiated, maintained, and propagated in vitro according to the manufacturer’s instructions. Briefly, RAW 264.7 cells were grown in Dulbecco’s Modified Eagle’s medium supplemented with fetal bovine serum to a final concentration of 10%. Cells were incubated at 37 °C in a 5% CO_2_ atmosphere at 95% humidity. For subculture, cells were scraped and centrifuged before seeding at a density of 5 × 10^4^ cells/mL (0.8 × 10^4^ cells/cm^2^). Cell treatments involved exposing cultured RAW 264.7 cells at 60–70% confluence to Si/SiO_2_ QDs at concentrations of 5 and 15 μg/mL for all assays, except for the MTT assay, where additional QD doses of 25 and 100 μg/mL were also employed. The cells were exposed to the QDs for different time intervals of 6, 12, and 24 h. To prepare the QDs for cell treatments, the QDs were suspended in cell culture media and carefully sonicated and dispersed using an ultrasonic processor (Hielscher UP50H, Hielscher Ultrasonics GmbH, Teltow, Germany) before use. 

### 2.3. Cell Viability and Proliferation Assessment

**Figure 1:**
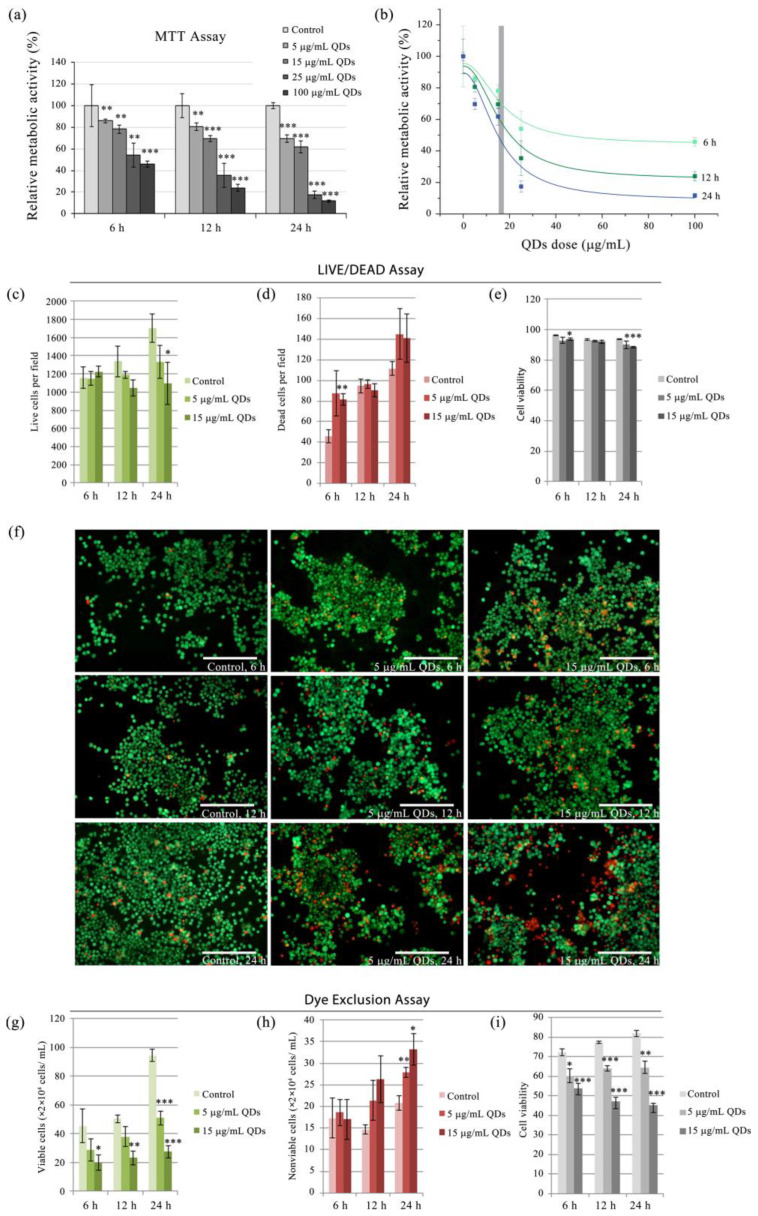
Cell proliferation and viability of murine RAW 264.7 macrophages exposed to Si/SiO_2_ QDs. (**a**) The cell metabolic activity revealed by MTT assay; (**b**) dose-response curves highlighting the IC50 doses; (**c**) the number of live cells estimated by LIVE/DEAD assay; (**d**) the number of dead cells estimated by LIVE/DEAD assay; (**e**) cell viability calculated from cell counts obtained from integration of specific fluorescence signals in LIVE/DEAD fluorescence micrographs; (**f**) representative examples of fluorescence micrographs of LIVE/DEAD stained RAW 264.7 cells in culture, exposed to 5 and 15 μg/mL QDs for 6, 12, and 24 h, along with the corresponding controls, with live cells stained in green and dead cells stained in red. Bar represents 200 μm; (**g**) trypan blue dye exclusion live assay cell counts; (**h**) trypan blue dye exclusion live assay cell counts; (**i**) cell viability calculated based on trypan blue dye exclusion assay. Data are presented as means ± SD. A student’s *t*-test was calculated to assess statistical significance relative to controls: * *p* ≤ 0.05, ** *p* ≤ 0.01, *** *p* ≤ 0.001.

Cell growth was evaluated using the MTT assay [32]. This method utilizes 3-(4,5-dimethylthiazol-2-yl)-2,5-diphenyltetrazolium bromide (MTT), which is permeable to normal cell membranes and is reduced intracellularly, forming water-insoluble formazan crystals, which are subsequently dissolved in isopropanol and quantified spectrophotometrically at 595 nm. For the MTT assay, cells were seeded in 24-well plates at a density of 10^5^ cells/mL and exposed to concentrations of 0, 5, 15, 25, or 100 μg QDs/mL for 6, 12, or 24 h. Subsequently, cells were incubated with 0.5 mg/mL MTT in culture medium for 2 h, and formazan crystals were dissolved in isopropanol and quantified at 595 nm using a TECAN GENios multireader, instrument serial no. 12900400226 (TECAN Group Ltd., Männedorf, Switzerland), equipped with Magellan v7.0 software. The MTT assay is often used as a measure of cellular proliferation because the MTT dye is metabolized by metabolically active cells and does not reflect the unviable part of the cell population. Using Origin Pro 8 software (OriginLab Corporation, Northampton, MA, USA), we analyzed the relationship between cell metabolic activity, assessed by MTT assay (Figure 1a), and the concentration of QDs. The data were fitted to the equation described below to determine the IC50 doses at each time interval. The IC50 represents the concentration at which the fitted curve reaches 50% inhibition of the metabolic activity level observed for each time interval. The resulting dose-response fitted curves are illustrated in Figure 1b.

$$y=START+(END-START)\frac{x^{n}}{k^{n}+x^{n}}$$

where y = relative metabolic activity (% of control); START = optical density at 595 nm in control cells; END = minimum optical density at 595 nm in QDs exposed cells for a given time interval; x = QDs concentration; n = Hill coefficient; and k = IC50. 

The cellular viability was evaluated using the LIVE/DEAD kit (Invitrogen, Carlsbad, CA, USA). This method allows simultaneous visualization, under a fluorescence microscope, of live, apoptotic, and dead cells using the calcein-AM (acetomethyl) and ethidium bromide dyes. Live cells are highlighted due to the ubiquitous presence of intracellular esterases, which enzymatically transform the non-fluorescent calcein-AM compound that can cross the intact cell membrane into a highly fluorescent compound called calcein (ex/em 495 nm/515 nm). Ethidium bromide cannot penetrate the intact cell membrane, but it can penetrate the compromised membrane of necrotic cells as well as that of apoptotic cells. After entering the cell, ethidium bromide intercalates between the nitrogenous bases of nucleic acids and increases its fluorescence by about 40 times (ex/em 495 nm/635 nm). To perform the LIVE/DEAD test, cells were seeded on 24-well plates and treated with different concentrations of silicon QD suspension. After 6, 12, and 24 h of treatment, the culture medium was removed and replaced with 200 μL of LIVE/DEAD solution optimized for the murine macrophage cell type (culture medium without fetal serum, containing 8 nM ethidium bromide and 2.4 μM calcein-AM). Cells were incubated for 40–50 min at 37 °C, then washed twice with phosphate-buffered saline (PBS) buffer and observed under an Olympus IX71 fluorescence microscope equipped with a TRITC/DAPI/FITC triple filter. Viable cells appeared green, while dead cells were stained red with ethidium bromide. The acquired images were subjected to pre-processing and quantification using ImageJ 1.53e software (National Institutes of Health, Bethesda, MD, USA) [33], which involved background subtraction, channel splitting, brightness and contrast adjustment, automatic counting, and measurement of calcein and ethidium bromide-stained cells. The data were exported to Microsoft’s Excel software, version 14.0.7113.5005 (Microsoft Corporation, Redmond, WA, USA), for statistical evaluation and representation.

The viability of cells was also assessed using the trypan blue exclusion assay. Cells were harvested and resuspended in PBS, and 0.4% trypan blue dye was added to the suspension. After incubation at room temperature for 5 min, the stained cells were visualized under a light microscope. Live cells with intact cell membranes exclude the dye and appear translucent, while dead cells with compromised membranes take up the dye and appear blue. The percentage of viable cells was determined by counting the number of unstained (viable) and stained (non-viable) cells in multiple fields using an inverted microscope. The experiment was repeated three times.

### 2.4. Cell Lysate Preparation

For obtaining cell lysates, the RAW 264.7 macrophages were seeded in 25 cm^2^ at a density of 10^5^ cells/mL and grown in Dulbecco’s Modified Eagle’s Medium, supplemented with fetal bovine serum to a final concentration of 10%. Cells were incubated at 37 °C in a 5% CO_2_ atmosphere at 95% humidity until they reached 60–70% confluence. Control cell media was changed to a fresh growing medium at the moment of QDs exposure, and cells were harvested at 6-, 12-, or 24-h intervals. Control cell media was changed to a fresh growing medium at the moment of QDs exposure and harvested at 6-, 12-, or 24-h intervals. Used culture media were also collected for extracellular LDH activity and cytokine level assessment. After in vitro treatments, the nanoparticle-containing medium was removed, and cells were washed with 1–2 mL of PBS. The cells were then mechanically detached using a cell scraper and centrifuged at 1500 rpm for 10 min at 18 °C. The resulting cell pellet was resuspended in a volume of PBS based on cell number and downstream analyses. The cell homogenate was sonicated three times for 30 s each on ice and then centrifuged at 3000 rpm for 10 min at 4 °C. The resulting supernatant (cell lysate/total protein extract) was frozen at −80 °C and later used for biochemical and immunological analyses. 

### 2.5. Total Protein Quantification

For protein concentration determination of the cell lysates, the Bradford protein assay kit (Bio-Rad) was used according to the manufacturer’s instructions. Optical density was measured at 595 nm using a TECAN GENios multireader (TECAN Group Ltd., Männedorf, Switzerland) equipped with Magellan v7.0 software, and a standard curve was constructed using bovine serum albumin (BSA).

### 2.6. Evaluation of Lactate Dehydrogenase Activity in the Culture Medium

To evaluate cellular membrane damage, the amount of lactate dehydrogenase (LDH) released into the culture medium was measured using the in vitro Toxicology Assay kit (Lactic Dehydrogenase) from Sigma (Sigma-Aldrich, St. Louis, MO, USA), following the manufacturer’s instructions. The kit employs a colorimetric method that relies on the reduction of NAD^+^ by LDH, leading to the formation of a colored formazan derivative. Cells were grown in 25 cm^2^ flasks and exposed to QD suspension, and the culture medium was collected and processed to obtain a cell-free supernatant. The culture medium was supplemented with 10% heat-inactivated fetal serum, which was used as a blank to correct for any background LDH activity. The corrected absorbance values were used to calculate the amount of LDH released into the medium. As LDH levels in the medium will be influenced by the number of cells present, we normalized them to the total number of cells determined by the dye exclusion assay. 

### 2.7. Intracellular Superoxide Anion Assessment

To quantitatively evaluate the intracellular level of reactive oxygen species (ROS), the superoxide anion was determined using nitroblue tetrazolium (NBT), a compound that can penetrate the plasma membrane and is reduced to an insoluble blue formazan by the superoxide anion. Cells cultured in 25 cm^2^ flasks were treated with QD suspension for 6, 12, and 24 h. At the end of each treatment interval, cells were scraped and centrifuged at 1500 rpm for 5 min at 18 °C. The cellular pellet was resuspended in 1 mL of PBS, homogenized, counted, and pelleted again. The pellet was resuspended in 0.5 mL of serum-free culture medium supplemented with 0.5 mg/mL NBT. Cells were subsequently incubated for one hour at 37 °C in a water bath. After incubation, cells were pelleted (1500 rpm, 5 min, 18 °C), and the pellet was solubilized in 200 μL of DMSO. To solubilize all formazan crystals, the suspension was sonicated for 30 s. The optical density was measured at a wavelength of 520 nm in a 96-well microplate using a TECAN GENios multireader (TECAN Group Ltd., Männedorf, Switzerland) equipped with Magellan v7.0 software.

### 2.8. Malondialdehyde Assay

Malondialdehyde (MDA) quantification was performed to assess lipid peroxidation using a method originally described by Del Rio et al. [18] that employs thiobarbituric acid (TBA) as the reactive substance. MDA-TBA adducts generated by the reaction between MDA present in the biological sample and TBA at 37 °C can be measured fluorimetrically. A standard solution of 1 μM MDA was used to construct a calibration curve. After adding 700 μL of 0.1 M HCl to tubes containing the standard solution and the appropriately diluted cellular lysate samples (200 μL), the mixture was homogenized and incubated for 20 min at room temperature. Next, 900 μL of 0.025 M TBA solution was added, and the mixture was incubated for 65 min at 37 °C to form TBA-MDA products. Fluorescence readings were obtained using a Jasco FP-750 fluorometer (Jasco Inc., Easton, MD, USA) with excitation and emission wavelengths of 520 nm and 549 nm, respectively. The degree of lipid peroxidation was assessed by calculating the MDA concentration relative to the protein concentration, and the results were expressed as μmoles MDA/mg protein.

### 2.9. Enzyme Activity Assessment

The superoxide dismutase (SOD) assay is based on the inhibition of NADH oxidation by SOD [34]. Beta-mercaptoethanol autooxidizes in the presence of EDTA and MnCl_2_, generating superoxide anions that oxidize NADH to NAD^+^, while SOD catalyzes the conversion of superoxide anions into molecular oxygen and hydrogen peroxide. In the presence of SOD, there is competition between the consumption of superoxide anions by SOD and the oxidation of NADH. After a 5-min pre-incubation period at 37 °C, the enzymatic reaction was initiated by adding β-mercaptoethanol solution and monitored for 20 min at 340 nm. The reaction medium contained 20 µL of cell lysate in a reaction mixture with a final volume of 213 µL and final concentrations of 9.4 mM β-mercaptoethanol, 5 mM triethanolamine-diethanolamine buffer, 0.25 mM EDTA, 0.125 mM MnCl_2_, and 0.15 mM NADH. The readings were taken relative to a blank control without cell lysate. The absorbance readings were obtained using a TECAN GENios multi-reader (TECAN Group Ltd., Männedorf, Switzerland) equipped with Magellan v7.0 software. 

Catalase (CAT) activity was measured using the spectrophotometric method described by Aebi [35]. The decrease in optical density at 240 nm was monitored for 1 min after adding the diluted lysate to a reaction mixture containing 76 mM potassium phosphate buffer (pH 7.1) and 22.95 mM H_2_O_2_ in a final reaction volume of 1000 µL. Absorbance readings were taken every 15 s using a PerkinElmer Lambda 25 UV/Vis spectrophotometer (PerkinElmer, Waltham, MA, USA).

GPX catalyzes the reduction of H_2_O_2_ or other peroxides, particularly lipids, in a reaction that involves GSH oxidation. The GPX activity was determined in a reaction medium containing an excess of glutathione reductase, which catalyzes the reduction of GSSG while converting NADPH to NADP^+^ [36]. The rate of this reaction was quantitatively determined by measuring the absorbance at 340 nm. The process of NADPH oxidation was monitored for 5 min at 25 °C after adding 10 µL of tert-butyl hydroperoxide (7 mM) to the reaction mixture consisting of 10 µL of diluted cell lysate and the reagent concentration of 70 μM tert-butyl hydroperoxide, 100 mM Tris/HCl buffer, 5 mM EDTA, 0.02 mM GSH, 120 μM NADPH, and 0.1–0.3 units glutathione reductase in a final reaction volume of 1000 µL. Enzymatic activity was calculated using the molar extinction coefficient of 6.22 × 10^3^ M^−1^ cm^−1^ for NADPH. The analysis was performed using a PerkinElmer Lambda 25 UV/Vis spectrophotometer (PerkinElmer, Waltham, MA, USA).

Gluthatione S-transferase (GST) activity was determined using the method described by Habig et al. [37]. The method uses the electrophilic substrate 1-chloro-2,4-dinitrobenzene (CDNB), whose conjugate with reduced glutathione absorbs at 340 nm. The increase in absorbance at 340 nm was monitored for 5 min at 25 °C after adding 50 µL of cell lysate to a reaction mixture containing 20 mM potassium phosphate buffer (pH 7.1), 0.5 mM CDNB, and 2 mM GSH. Enzymatic activity was calculated using the molar extinction coefficient of 9.6 × 10^3^ M^−1^ cm^−1^. The analysis was performed using a PerkinElmer Lambda 25 UV/Vis spectrophotometer (PerkinElmer, Waltham, MA, USA). All enzyme activities were normalized to the protein concentration of the cell lysate and expressed as U/mg protein. 

The activity of caspase-1 was assessed using the Caspase-1/ICE Colorimetric Assay kit (Biovision, Milpitas, CA, USA) according to the manufacturer’s instructions. The method is based on the spectrophotometric detection of the chromophore p-nitroanilide (p-NA) after its cleavage from the labeled substrate YVAD-pNA by caspases that specifically recognize the tetrapeptide Tyr-Val-Ala-Asp. Cell lysates, with previously determined protein concentrations, were adjusted to 200 μg protein/50 μL. Dithiothreitol (DTT), provided in the kit as a 1 M solution, was diluted to a 10 mM concentration. To each sample (25 μL), an equal volume of reaction buffer containing 10 mM DTT and 5 μL of YVAD-pNA substrate (final concentration of 200 μM/reaction) was added. The homogeneous reaction mixture was incubated for two hours at 37 °C, and the absorbance was read at 405 nm. The background absorbance was subtracted from the treated and untreated (control) samples (using a similar procedure with the exception that cell lysate was not used but instead phosphate-buffered saline was used). The relative change in caspase-1 activity was calculated by normalizing treated samples to untreated ones.

### 2.10. Western Blotting

Western blot analysis was performed to detect any changes in protein expression levels induced by Si/SiO_2_ QDs treatment. Briefly, total protein samples (30 μg/lane) were denatured at 95–100 °C in the presence of denaturing sample loading buffer for 5 min and separated by sodium dodecyl sulfate–polyacrylamide gel electrophoresis using a 10% polyacrylamide gel. Membranes were blocked with the blocking solution provided in the Western Breeze Chromogenic Immunodetection System (Invitrogen, Carlsbad, CA, USA) according to the manufacturer’s instructions. The separated proteins were transferred to PVDF membranes using a wet transfer method. The membrane was incubated with primary antibodies against the target proteins of interest. Primary antibodies for B-cell lymphoma 2 (Bcl-2) and Beclin 1 detection were of murine origin (Bcl-2 (C-2):sc-7382, dilution 1:500; BECN(G11):sc-48381, dilution 1:1000) and Nrf-2 rabbit polyclonal antibody (H-300 SC-13032, dilution 1:1000) from Santa Cruz Biotechnology (Dallas, TX, USA). Heat shock proteins were assessed with anti-HSP-60 (1:1000 dilution, mouse monoclonal IgG; catalog no. 386028, Calbiochem, San Diego, CA, USA), the monoclonal HSP70 antibody (NCL-HSP70, clone 8B11), and the monoclonal HSP90 antibody (NCL-HSP90, clone JPB24) (Novocastra, Newcastle upon Tyne, UK). β-actin, a housekeeping protein, was targeted with a mouse monoclonal anti-β-actin A1978 antibody (Sigma-Aldrich, St. Louis, MO, USA), followed by secondary antibodies conjugated to alkaline phosphatase. The proteins were visualized using a chromogenic detection system, and the resulting bands were quantified using densitometry. The Western blotting analysis was performed using the anti-mouse or anti-rabbit WesternBreeze™ Chromogenic Kit (Invitrogen, Carlsbad, CA, USA), according to the manufacturer’s instructions. The revealed membranes were analyzed using the Vilbert Lourmat image acquisition system, BioCapt 12.6 (Vilbert Lourmat, Marne-la-Vallée, France), and ImageJ 1.53e software (National Institutes of Health, Bethesda, MD, USA) [33].

### 2.11. Inflammatory Biomarkers Assessment

The capacity of Si QDs to induce nitric oxide production in murine RAW 264.7 macrophages was evaluated indirectly by measuring nitrite. Nitrite is the final and stable compound produced by the interaction of nitric oxide with various cellular molecular targets. Nitric oxide is a cytotoxic and antimicrobial agent secreted by macrophages and has a half-life of several seconds. In the presence of oxygen in an aqueous medium, nitric oxide reacts with itself, generating nitrogen oxide intermediates, which then decompose to form nitrite and nitrate. The method involves reducing NO_3_^−^ to NO_2_^−^ and then using the Griess reaction to determine NO_2_^−^ [38]. The Griess reaction is a two-step diazotization process where N_2_O_3_, derived from nitrous acid, reacts with sulfanilamide to produce a diazonium ion. The diazonium ion couples with N-(1-naphthyl)ethylenediamine, forming a chromophoric azo product that absorbs strongly at 540 nm. The reagents used were a stock solution of 100 mM NaNO_2_, 10% N-(1-napthyl)ethylenediamine, and 1% sulfanilamide solution in 5% H_3_PO_4_. Immediately before starting the protocol, a mixture of the 10% N-(1-napthyl)ethylenediamine solution and the 1% sulfanilamide solution was prepared in a 1:1 ratio. Nitrite quantification was performed using a standard curve obtained from serial dilutions of the NaNO_2_ stock solution in culture medium with 10% fetal bovine serum. For each analysis, 80 μL of the collected culture medium or the diluted solutions prepared for the standard curve were added to 80 μL of N-(1-napthyl)ethylenediamine:sulfanilamide, homogenized, and subsequently the optical density was immediately read at 540 nm. The nitrite concentrations in the supernatants were normalized to the number of cells in each sample.

Cyclooxygenase activity levels are relevant for inflammatory response evaluation as they are enzymes involved in the synthesis of prostaglandins. In this study, the Cayman COX Fluorescent Assay kit (Cayman Chemical, Ann Arbor, MI, USA) was used to determine the enzymatic activity of COX-1 and COX-2, following the manufacturer’s instructions. This assay is based on a fluorescent method that measures the peroxidase activity of COX, which catalyzes the oxidation of ADHP (10-acetyl-3,7-dihydroxyphenoxazine) to produce a highly fluorescent compound, resorufin. The assay uses specific compounds for each COX isoform provided in the kit (DuP-697—a specific inhibitor of COX-2; SC-560—a specific inhibitor of COX-1). In our case, the selective inhibitor of COX-1 used in the kit (SC-560) did not allow for the differentiation of the activities of the two COX isoforms in RAW 264.7 cell lysates, as it does not inhibit COX-1 activity in this cell line, which is in line with Brenneis et al., the authors who previously found that SC-560 does not inhibit COX-1 in RAW 264.7 cells [39]. For the assay procedure, protein extracts were diluted in the working buffer (100 mM Tris-HCl, pH 8) to concentrations ranging from 0–2 μM for the quantification of COX activity. The diluted samples and the standard curve were pipetted into a 96-well black opaque fluorescence plate in duplicate. Fluorescence was immediately read at Ex 535 nm/Em 590 nm using a fluorimeter. The specific COX-2 activity was calculated by extrapolating the fluorescence values of the samples onto the standard curve and expressed as U/mg total protein. 

Prostaglandin E2 (PGE2) is an inflammation mediator that regulates various physiological functions. COX converts arachidonic acid to PGH2, which is then turned into PGE2 by PGES-1, PGES-2, or cPGES [40]. To measure PGE2 released into the culture medium following the exposure of RAW 264.7 cells to Si/SiO_2_ QDs, a competitive Human Prostaglandin E2 ELISA kit (Invitrogen, Carlsbad, CA, USA) was used. The method is based on the competition between PGE2 present in the culture medium and the tracer represented by PGE2 conjugated with alkaline phosphatase for a limited amount of specific monoclonal anti-PGE2 antibodies. The equilibration takes place in a 96-well plate pre-treated with polyclonal goat antibodies that bind to all mouse monoclonal anti-PGE2 antibodies that are added (provided in the kit). After incubation, the supernatants were aspirated, and the plate was washed with 250 μL of wash solution five times. Furthermore, a solution of para-nitrophenyl phosphate, a substrate for alkaline phosphatase, was added. The enzymatic reaction product absorbs at 412 nm and is inversely proportional to the amount of PGE2 in each sample. The PGE2 concentration was calculated by extrapolating the absorbance of samples onto the standard curve.

Cytokines were assessed in both cell lysates and cell culture media with the Bio-Plex Pro Mouse Cytokine 23-plex Assay. The assay was performed according to the manufacturer’s instructions (Bio-Rad Laboratories, Inc., Hercules, CA, USA) with slight modifications. In brief, the assay involved the preparation of a series of standards with known concentrations of cytokines. Cell lysate protein concentrations were equalized to 9.2 mg/mL, while cell media was used undiluted. For the assay procedure, magnetic beads coated with antibodies specific for each cytokine were conjugated with the corresponding capture antibodies and were subsequently incubated with the standards or samples for 30 min at room temperature on an orbital shaker. After incubation, the beads were washed three times with the wash buffer provided by the manufacturer, and detection antibodies were added to each well and incubated for 30 min at room temperature on a shaker. Streptavidin-phycoerythrin was added to each well and incubated for 10 min at room temperature with mild homogenization on an orbital shaker. Finally_,_ the beads were washed three times with the wash buffer provided by the manufacturer. The beads were resuspended in assay buffer and analyzed using the Bio-Plex MAGPIX Multiplex Reader (Bio-Rad Laboratories, Inc., Hercules, CA, USA). Data analysis was performed using the Bio-Plex Manager software version 6.2 (Bio-Rad Laboratories, Inc., Hercules, CA, USA). The Bio-Plex Pro Mouse Cytokine 23-plex Assay allowed for the simultaneous detection of 23 different pro- or anti-inflammatory cytokines, interleukins, chemokines, and growth factors: IL-1α, IL-1β, IL-2, IL-3, IL-4, IL-5, IL-6, IL-9, IL-10, IL-12 (p40), IL-12 (p70), IL-13, IL-17A, Eotaxin, G-CSF, GM-CSF, IFN-γ, KC, MCP-1 (MCAF), MIP-1α, MIP-1β, RANTES, and TNF-α. The assay was performed in triplicate for each sample. Cytokines below the range of detection were assigned a value of the lower limit of quantitation of the assay for quantitative analysis. Cytokines that exceeded the detection range (labeled OOR> in Supplementary Table S1) were deemed beyond the quantifiable limit of the assay and reported as such.

### 2.12. Autophagosome Labeling

Cellular autophagy was determined using the Autophagy/Cytotoxicity Dual Staining kit from Cayman (Ann Arbor, MI, USA). This kit uses MDC (monodansylcadaverine), a fluorescent compound that is incorporated into the multilamellar vesicles of autophagic vacuoles through an ion capture mechanism as well as through its interaction with membrane lipids. Propidium iodide (PI) was used as a marker of cell death.

Cells were seeded at a density of 10^5^ cells/well in 24-well plates, and after 48 h of growth, they were treated with QDs. At the end of each 6-, 12-, and 24-h QD treatment interval, the culture plates were centrifuged (400× *g*, 2 min, 25 °C), and the medium was carefully aspirated to avoid disturbing detached cells (this operation was repeated before each aspiration procedure in the wells to avoid also removing dead or detached cells from the substrate). Moreover, 100 μL of PI solution (diluted according to the kit instructions, 1000-fold) was added to each well. After incubating for 10 min at room temperature, the PI was removed, and the cells were washed with 100 μL of wash buffer/well and treated with 100 μL of MDC solution (diluted according to the kit instructions, 1000-fold) per well for 10 min at 37 °C. After this interval, the MDC solution was replaced with buffer (supplied in the kit), and the cells were immediately visualized under the Olympus IX71 fluorescence microscope (TRITC/FITC/DAPI triple filter). PI highlighted dead cells in red, while MDC highlighted the autophagic vacuoles in blue-green. ImageJ 1.53e software (National Institutes of Health, Bethesda, MD, USA) [33] was used for image pre-processing and quantification of the areas stained with MDC. The ImageJ analysis involved background subtraction, channel splitting, brightness and contrast adjustment, automatic counting, and measurement of MDC-stained cells. The data were exported to Microsoft’s Excel software, version 14.0.7113.5005 (Microsoft Corporation, Redmond, WA, USA), for statistical evaluation and representation.

### 2.13. Statistical Analysis

The student’s *t*-test was used to analyze the results and determine if there was a significant difference between the experimental groups and the appropriate control group (*p* ≤ 0.05 was considered statistically significant). The data are presented as the mean ± standard deviation from three independent experiments. They are expressed either as absolute values or relative to the control levels, depending on the specific case indicated in the figure legends. Pearson’s correlation test was used to examine the relationship between variables, assess the strength of their associations, and determine the significance of the observed correlations. The correlation coefficient, denoted as “r,” ranges from −1 to 1, where −1 indicates a perfect negative correlation, 1 indicates a perfect positive correlation, and 0 indicates no correlation. The probability (*p*-value) associated with the observed correlation coefficient was determined using the t-distribution formula:

$$t=r\times \sqrt{\left(n-2\right)}/\sqrt{1-r^{2}}$$

where t represents the t-value associated with the correlation coefficient (r), r is the calculated Pearson correlation coefficient, and n is the number of data points. All the statistical analyses were performed using Microsoft Excel software version 14.0.7113.5005 (Microsoft, Redmond, WA, USA).

## 3. Results and Discussion

### 3.1. Cytotoxic Assessment of Si/SiO_2_ Quantum Dots in RAW 264.7 Cells

The text continues here. The QDs concentration, which diminished by 50% the metabolic activity of cells (IC50) was calculated from data obtained by MTT assay, performed using doses of 5, 15, 25, 50, and 100 μg/mL at exposure intervals of 6 h, 12 h, and 24 h. The relative metabolic activity of RAW 264.7 cells exposed to varying doses of QDs (5 μg/mL, 15 μg/mL, 25 μg/mL, and 100 μg/mL) decreased in a dose- and time-dependent manner (Figure 1a). The IC50 doses corresponding to the 6 h, 12 h, and 24 h QDs exposure intervals were 16.5, 16.5, and 15.3 μg/mL, respectively (Figure 1b), indicating that both QDs concentration and exposure time influenced cytotoxicity, reaching a decrease of 88% viability compared to control at a dose of 100 μg/mL at the 24 h exposure (Figure 1a,b). 

This response to Si/SiO_2_ QDs differs from the one reported previously in human hepatocarcinoma cells, which, at doses three times higher and longer exposure periods, had a maximum metabolic activity reduction of 50% [17]. Similarly, upon exposure of MRC-5 fibroblast cells to these QDs, cytotoxic effects were observed for exposure intervals longer than 24 h and in particular for concentrations over 100 μg/mL QDs [18]. Surprisingly, the effects of 25 μg/mL and 100 μg/mL QDs doses on RAW 264.7 cells metabolic activity appeared comparable in intensity, particularly at the 12 h and 24 h time points. These observations suggest that there is a variation in the response to the QDs exposure among different types of cells, wherein the cytotoxicity toward macrophage-phenotype cells (RAW 264.7) is higher compared to other cell types. Given that the QDs used were the same in both cited papers and this study, the dissimilarities in the observed cytotoxic effects can be attributed to the variations in cell type. In this study, we utilized RAW 264.7 macrophages, while the previous papers employed HepG2 hepatocarcinoma cells and MRC-5 fibroblast cells. Each cell line possesses unique characteristics, including variances in receptor expression, intracellular signaling pathways, cellular responses, and biological roles, which can contribute to variations in the response to Si/SiO_2_ QDs. The complex interactions between QDs and different cell types pose challenges in translating results between in vitro and in vivo models. Moreover, the microenvironment and cell-cell interactions in vivo further complicate the response to QDs. These divergent findings highlight the importance of conducting extensive testing and considering cell type specificity when assessing the QDs safety.

For the evaluation of more subtle effects and the identification of the mechanisms of action involved in Si/SiO_2_ QDs cytotoxicity in RAW 264.7 macrophages, two nanoparticle concentrations were selected, namely the IC50 corresponding dose of 15 μg/mL and a lower dose of 5 μg/mL. The tolerance of macrophages to these doses of QDs was evaluated microscopically by assessing viability using the LIVE/DEAD assay (Figure 1c–f). Based on image analysis, the LIVE/DEAD assay revealed a reduction in the number of viable cells, which exhibited an inverse relationship with the QD dose and duration of exposure; however, this was significant statistically only at 24 h exposure to 15 μg/mL Si/SiO_2_ QDs (Figure 1c). In addition, the increase in the number of nonviable cells (stained in red with PI) was noted at 6 h and 24 h of exposure, with only the shortest interval being statistically significant at the 15 μg/mL Si/SiO_2_ QDs dose (Figure 1d). A dye exclusion assay was performed to further verify these observations. In this case, given that cell counting was less prone to errors caused by overlapping cells, the antiproliferative component of the QDs was more pronounced and significant statistically at all the time intervals for the 15 μg/mL QDs dose and at 24 h for the 5 μg/mL QDs dose, respectively (Figure 1g). The number of nonviable cells, considered cells with membranes unable to exclude the dye, increased in a dose- and time-dependent manner; however, it was statistically significant only at the 24-h exposure interval for both QD doses (Figure 1h). This suggests that the reduction of metabolic activity described by the MTT assay could be explained not only by cytotoxic effects but also by an antiproliferative outcome of macrophages exposed to QDs. Cell viability (calculated as percent of viable cells out of the total cells counted) was independently determined based on LIVE/DEAD assay fluorescence micrograph analysis and dye exclusion assay with subsequent hemocytometer manual cell counting. The relative cell viability obtained with the dye exclusion assay at the 6-, 12-, and 24-h time intervals of exposure to 5 μg/mL QDs was 83.9%, 79.0%, and 79.8%, respectively, while the exposure to 15 μg/mL QDs dose reduced cell viability to 74.4%, 58.0%, and 54.9% compared to that of the control cells after 6, 12, and 24 h, respectively (Figure 1i, which shows absolute cell viability levels). On the other hand, for the same time intervals, LIVE/DEAD assay viability data yielded relative cell viabilities of 96.6%, 99.1%, and 95.9% for the dose of 5 μg/mL and 97.4%, 98.5%, and 94.3% for the 15 μg/mL QDs dose (absolute LIVE/DEAD assay viability levels are presented in Figure 1e). 

The cell viability data obtained from both the dye exclusion assay and the live/dead assay show that exposure to Si/SiO_2_ QDs at concentrations of 5 μg/mL and 15 μg/mL resulted in reduced cell viability compared to the control. Analysis of cellular morphology revealed changes such as transparent cytoplasm, mostly intact nuclear structure, and increased cell volume with extensive vacuolations (Supplementary Figure S1), which correspond to cell architecture changes associated with necrotic or oncotic cell death previously described [41,42,43,44]. It is, however, important to note that these morphological observations are not definitive and warrant further exploration in order to elucidate the specific type of cell death induced by Si/SiO_2_ QDs in RAW 264.7 murine macrophages. 

**Figure 2:**
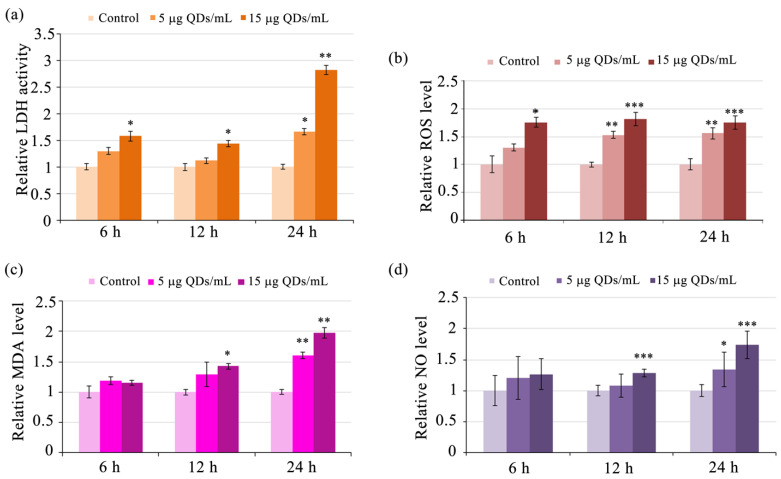
Oxidative stress indicators in RAW 264.7 cells exposed to Si/SiO_2_ QDs. (**a**) Extracellular LDH activity; (**b**) ROS levels; (**c**) MDA levels; and (**d**) NO levels in whole cell lysates. Data are presented as means ± SD. A student’s *t*-test was calculated to assess statistical significance relative to controls: * *p* ≤ 0.05, ** *p* ≤ 0.01, *** *p* ≤ 0.001.

To further investigate the relationship between Si/SiO_2_ QDs and cell death, the activity of lactate dehydrogenase (LDH) was measured in murine macrophages RAW 264.7 exposed to QDs at different time intervals. LDH activity was significantly higher in cells exposed to 15 μg/mL QDs compared to the control group at all intervals (*p* < 0.05), indicating that QD exposure can lead to cell membrane damage (Figure 2a). The highest LDH activity was observed at the 24-h interval for both QD concentrations, for 5 μg/mL QDs by 1.66-fold (*p* = 0.021) and 15 μg/mL QDs by 2.83-fold (*p* = 0.003), respectively (Figure 1a). A significant negative correlation between LDH activity and cell viability (Pearson’s correlation coefficient r = −0.7569, *p* = 0.010) was observed, which indicated that membrane leakage is one of the QD-induced deleterious effects that could contribute to cell death through a necrotic mechanism. The Pearson correlation coefficients revealed strong and positive correlations between LDH fold changes and those of MDA (r = 0.8730, *p* = 0.002), NO (r = 0.9894, *p* < 0.001), and ROS (r = 0.8200, *p* = 0.005), suggesting that oxidative stress might be a potential mechanism by which Si/SiO_2_ QDs induce macrophage membrane leakage. 

ROS and MDA levels in murine macrophages RAW 264.7 exposed to Si/SiO_2_ QDs were significantly higher in cells exposed to QDs compared to the control group at all intervals for the 15 μg/mL QDs dose and only at the 12- and 24-h intervals for the 5 μg/mL QDs concentration. In cells exposed to the 15 μg/mL QDs dose, the fold increases in ROS levels relative to controls ranged from 1.75 to 1.81-fold depending on the QDs concentration and exposure time (*p* < 0.05 at 6 h and *p* < 0.001 for 12 and 24 h) (Figure 2b). Similarly, MDA levels were significantly higher in the cells exposed to QDs compared to the control group, and these increases were statistically significant at the 12-h exposure interval to 15 μg/mL of QDs (*p* < 0.05) and the 24-h interval for both QD concentrations (*p* < 0.01), indicating that these QDs can lead to oxidative damage in macrophages in a dose-dependent manner (Figure 2c). Notably, although the MDA levels were consistently higher than those of controls, the fold increase in MDA levels in cells exposed to the 15 μg/mL doses did not show a tendency to increase with exposure time. 

Nitric oxide (NO) plays a fundamental role in inflammation by acting as a toxic agent towards pathogens as well as an immunoregulator and an inducer or suppressor of immune cell apoptosis. Macrophages produce NO through the activation of inducible nitric oxide synthase (iNOS) in response to various stimuli, such as bacterial endotoxins, cytokines, and nanoparticles. NO is a potent free radical that plays a critical role in immune responses by killing invading pathogens, tumor cells, and infected host cells. Excessive NO production can cause increased oxidative and nitrosative stress, DNA damage, dysregulation of energy metabolism, mitochondrial dysfunction [45], and calcium homeostasis, leading to cellular dysfunction, including the loss of membrane integrity [46]. In cultured chondrocytes, NO was also shown to be a mediator of sterile inflammation mediated by IL-1, leading to extracellular matrix dysregulation [47]. NO reacts with superoxide to form peroxynitrite, a very potent oxidant that can cause lipid peroxidation and protein oxidation, leading to membrane damage and cellular dysfunction [48]. 

In Si/SiO_2_ QDs-exposed cells, NO levels were consistently increased, with the 15 μg/mL dose generating statistically significant increases of 1.3- and 1.7-fold compared to control at the 12- and 24-h time intervals (*p* < 0.001), respectively (Figure 2d). The 5 μg/mL dose determined a 1.34-fold increase only at 24 h of exposure (*p* = 0.027). The Pearson correlation coefficient between cell viability and NO levels was r = −0.7928 (*p* = 0.007). 

This Si/SiO_2_ QD’s ability to induce NO synthesis was also shown in a fibroblast experimental model, which demonstrated an acute inflammatory response induced by Si/SiO_2_ QDs, mediated by the release of IL-6 and IL-8, during 72 h of exposure to 50 μg/mL QDs [31]. An important body of literature suggests that SiO_2_ nanoparticles (NPs) display similar toxicity to micrometric crystalline silica [49], and although the exact mechanisms are still unclear, their marked cytotoxicity towards macrophages, characterized by release of nitric oxide, tumor necrosis factor α (TNF-α) production, NF-kB and cyclooxygenase II activation, and induction of oxidative stress, has been proposed [50,51]. 

Normally, cells manage oxidative stress by activating antioxidant and detoxification enzymes through the action of the transcription factor known as nuclear factor erythroid-derived 2 related factor 2 (Nrf2). Consequently, cells may mitigate the consequences of stimuli that lead to necrosis and apoptosis [52,53]. 

### 3.2. Impact of Si/SiO_2_ Quantum Dots on the Inflammatory Response of RAW 264.7 Cells

**Figure 3:**
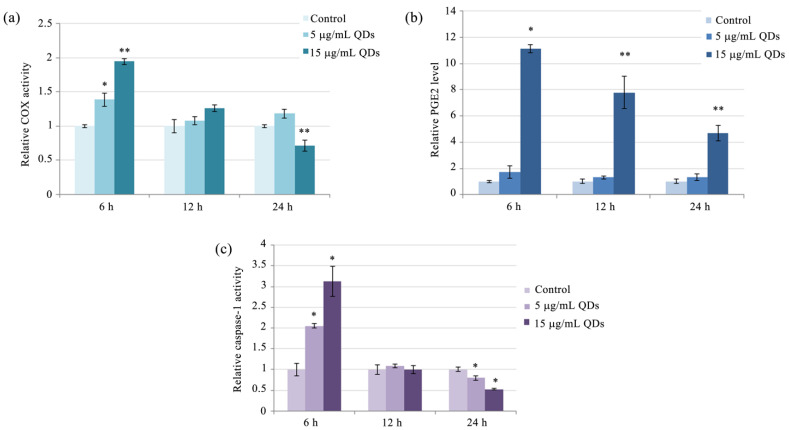
Inflammatory-related indicators in RAW 264.7 macrophages exposed to Si/SiO_2_ QDs. (**a**) Relative COX-2 enzyme activity level; (**b**) relative PGE2 levels; (**c**) relative caspase 1 activity levels. Data represent means ± SD. Statistically significant differences (student’s *t*-test) relative to control are indicated by * *p* ≤ 0.05; ** *p* ≤ 0.01.

Cyclooxygenases COX-1 and COX-2 are enzymes that are responsible for the production of prostaglandins (PGs), which are important for the regulation of immune and inflammatory responses. COX-2 is the inducible isoform, active in cells that have been stimulated with inflammatory mediators [54]. The COX-2 activity was significantly increased in a dose-dependent manner, reaching its maximum level at 6 h post-exposure. At the highest QDs dose (15 μg/mL), COX-2 activity levels were 1.9-fold higher than control cells. However, COX-2 activity gradually decreased over time, reaching similar levels to control cells by 12 h and below control levels at 24 h post-exposure. These findings suggest that Si/SiO_2_ QDs exposure can induce a transient increase in COX-2 activity in RAW 264.7 cells, which is followed by a decline over time (Figure 3a). As an outcome of this COX-2 activity pattern, the 15 μg/mL QDs dose had a marked effect on increasing PGE-2 levels, which peaked at the 6 h time point by over 11-fold higher than the levels registered in controls and subsequently continued to remain elevated, although overall the prostaglandin levels demonstrated a decreasing tendency (Figure 3b). 

Previous studies have highlighted a link between autophagy and the maturation of pro-inflammatory cytokines in the inflammasome of macrophages [55]. Autophagy promoted the degradation of pro-IL-1β, a precursor of the cytokine IL-1β that is cleaved by caspase-1 during inflammasome activation [56], generating an anti-inflammatory effect. A recent study has shown that silicone-based nanoparticles were able to induce inflammasome activation [57] in an in vitro cell model. Activation of the inflammasome in macrophages leads to the recruitment and assembly of multiple proteins, which in turn recruit the effector protease caspase-1. Caspase-1 cleaves pro-IL-1β and pro-IL-18 to their active forms, which are then secreted from the cell and promote inflammation and immune responses [58,59]. In RAW 264.7 cells, exposure to Si/SiO_2_ QDs induced a dose-dependent activation of caspase-1 activity, which, as in the case of the inducible COX isoform, peaked at the 6-h interval and thereafter decreased, even reaching levels lower than in control cells at the 24-h interval (Figure 3c). 

In this study, we also aimed to investigate the impact of Si/SiO_2_ QDs on RAW 264.7 cells in regards to the production of several cytokines in order to evaluate their potential proinflammatory effects. Cytokine levels were measured from both cell media and whole cell lysates using the Bio-Rad murine 23-plex assay, which allowed for the simultaneous measurement of 23 cytokines. Some cytokines exhibited constitutively low levels, irrespective of the QD treatments, while others displayed elevated levels and were unresponsive to the treatments (Supplementary Table S1). IL-2, IL-3, IL-4, IL-5, and IL-9 are generally associated with Type 2 adaptive immunity and antibody production, which are distinctive features of B lymphocytes rather than RAW 264.7 macrophages. RAW 264.7 macrophages are not typically involved in antibody production or the Type 2 immune response. The levels of IL-2, IL-3, IL-4, IL-5, and IL-9 cytokines were very similar in whole cell lysates and in the media and were found to be constitutively low. In both lysates and media, this group of cytokines ranged between 2.1 and 17.2 pg/mL, with SDs ranging between 0.8 and 5.8 pg/mL. Additionally, in RAW 264.7 cells exposed to Si/SiO_2_ QDs, IFN-γ, a potent cytokine that plays a crucial role in innate and adaptive immunity, was also expressed at constitutively low levels and was unresponsive to treatments (mean was 11.2 pg/mL and SD = 5.0 pg/mL). While RAW 264.7 macrophages are known to respond to IFN-γ challenge, previous studies have reported that they may not express significant levels of the cytokine themselves [60]. In a ddition, IL-17A is produced by various cell types, such as the T-helper 17 subset of CD4+ cells, Tc17CD8+ T cells, γδT cells, NK cells, and granulocytes [61], and is involved in promoting inflammation and defense against extracellular pathogens. The RAW 264.7 cell line is not typically recognized as a significant source of IL-17A cytokine production, and we found the levels of this cytokine to be unresponsive to QD exposure and expressed at constitutively low levels, both in cell lysates and in cell media (mean 6.6 pg/mL, SD = 3.8 pg/mL). 

MIP-1β and MCP-1 are chemokines involved in the recruitment and activation of monocytes, macrophages, and other immune cells to sites of inflammation. In our experimental conditions, we found their levels to be particularly elevated in the cell media, and regardless of the QD treatments, they were outside of the range of our detection method. The exact mechanisms behind the particularly high extracellular levels of MCP-1 and MIP-1β in RAW 264.7 cells, including control cells, require further investigation. Their presence in the extracellular environment, even in the absence of specific stimuli, may reflect the dynamic nature of immune cell responses and the inherent properties of RAW 264.7 macrophages.

**Figure 4:**
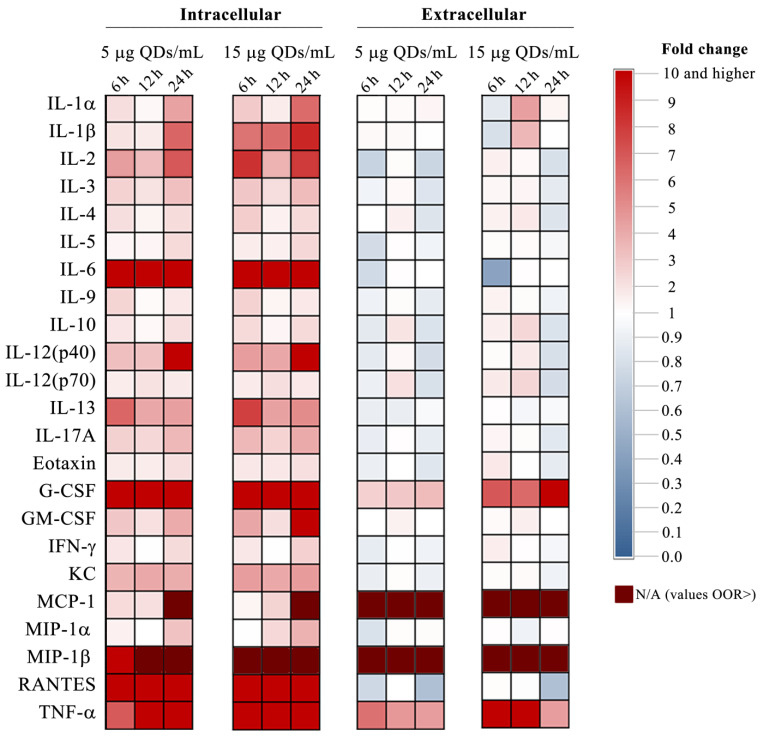
Heat-plot graph depicting the relative fold changes of a panel of 23 cytokines in QD-exposed RAW 264.7 macrophages relative to controls. The color key is shown on the right. All fold changes greater than 10-fold are represented by the same color. OOR > out of range, higher. The image was constructed based on the information presented in Supplementary Table S1, where the absolute values, SD, and statistical significance of the fold changes are available.

Moreover, in cell lysates from RAW 264.7 exposed for 6 h and 12 h to QDs, we observed very low levels of expression of the pro-inflammatory cytokines IL-1α and IL-1β. However, they were upregulated at the 24-h time point in a manner dependent on the QD dose applied. IL-1α levels in cell lysates were 14.7 pg/mL and 21.3 pg/mL after exposure to 5 and 15 μg Si/SiO_2_ doses, which represented 4.3-fold and 6.3-fold increases, respectively, relative to the control. IL-1β was also slightly upregulated and reached 12.2 pg/mL and 16.5 pg/mL after exposure to 5 and 15 μg Si/SiO_2_ doses, which represented 6.4-fold and 8.7-fold increases relative to the control (Supplementary Table S1). The levels of the mentioned cytokines in the cell media were found to be consistently low, except at the 12-h time point, where a transient, dose-dependent increase was observed (Figure 4 and Supplementary Table S1). This trend might be attributed to the shortened half-life of cytokines in the cell media, as our experimental setup did not involve continuous perfusion of the cells. 

Among the pro-inflammatory cytokines measured, the QDs treatment elicited a significant increase in the levels of IL-6, TNF-α, and RANTES, indicating their strong responsiveness to the treatment. Among these cytokines, IL-6 was exceptional, being increased in the cell lysate level over 2600-fold in cells treated with the 15 μg/mL Si/SiO_2_ QDs dose for 24 h, while no corresponding increase was observed in the cell media (Figure 4, Supplementary Table S1). Previously, in RAW 264.7 murine macrophages challenged with LPS, superparamagnetic iron oxide nanoparticles inhibited the release of the inflammatory cytokines IL-6 and TNF-α, resulting in an anti-inflammatory effect and attenuation of the immune and inflammatory response [61]. In our case, this effect is seen only in the case of IL-6, as extracellular fold changes of TNF-α were, with one exception, higher than the levels found in whole cell lysates (Supplementary Table S1). Increases in TNF-α levels in the cell media can have pleiotropic effects, including macrophage activation, increased production of ROS and NO, and induction of apoptosis or necroptosis in specific cell types [62]. Our results indicate that exposure of macrophages to silicone-based QDs can lead to the generation of ROS and NO, as evidenced by the data shown in Figure 2. Furthermore, microscopic evaluation of cells in culture seemed to indicate necrosis-like morphology (Supplementary Figure S1). Taken together, these findings suggest a possible TNF-α mediated mechanism of action for QD-induced citotoxicity in macrophage cells. 

IL-12 plays a crucial role in both the innate and adaptive immune responses against intracellular pathogens. It is a heterodimer produced mainly by macrophages and dendritic cells. IL-12 is essential for inducing T cell-dependent and independent activation of macrophages, among other immune cells. The genes that encode the two heterologous chains of IL-12, p40 and p35, are found on different chromosomes in both humans and mice. When combined, p40 and p35 form the biologically active IL-12, also known as p70 [63]. In RAW 264.7 cells exposed to silicone-based QDs, both cytokines increased in a dose- and time-dependent manner; however, IL-12 (p40) was more responsive, having more elevated levels (Supplementary Table S1).

The maximal RANTES concentrations were observed in whole cell lysates at the 24-h time point, with no apparent dose dependency. A concentration of 5 μg/mL QDs elicited the greatest recorded RANTES level at 189.5 ng/mL, while 15 μg/mL quantum dots resulted in a less pronounced upregulation, reaching 68.1 ng/mL. Nonetheless, under these conditions, the increase in RANTES levels was more than 3200 times higher than the control ones. In cell culture media, RANTES levels were relatively low, ranging between 48.5 and 195.4 pg/mL with a SD of 56.5. As consistent increases were only noted in the cell lysates but not in the cell media, we can assume either a delayed or impaired secretion of RANTES, which could have various outcomes, ranging from altered immune responses to cellular stress and the potential induction of cell death. 

Both granulocyte colony-stimulating factor (G-CSF) and granulocyte-macrophage colony-stimulating factor (GM-CSF) serve as essential regulators in the immune system, influencing the differentiation, proliferation, and functionality of granulocytes and macrophages, and have been considered to shift the phenotype of macrophages into a M1-like, pro-inflammatory polarization state [64]. Conversely, G-CSF predominantly targets the production and activity of granulocytes, specifically neutrophils [65]. The analysis of these two critical cytokines in macrophage cells exposed to Si/SiO_2_ QDs yielded noteworthy findings. For both cytokines, we observed dose-dependent and time-dependent increases in intracellular levels. Intriguingly, G-CSF secretion was detected at overall higher levels than GM-CSF, both in whole cell lysates and in the culture media (Supplementary Table S1). The findings of our study revealed a significant increase in intracellular G-CSF levels of over 16 × 10^3^-fold and in extracellular levels of up to 34-fold after 24 h exposure to 15 μg/mL Si/SiO_2_ QDs (the highest recorded increases among the 23-cytokine panel). GM-CSF levels showed no significant changes in the cell media (Figure 4 and Supplementary Table S1). This increase in G-CSF secretion might have profound implications at the organismal level, shedding light on the potential impact of Si/SiO_2_ QDs on the immune system and providing valuable direction for future research. Specifically, elevated levels of G-CSF can lead to the recruitment and activation of neutrophils at the site of infection or inflammation, potentially exacerbating the immune response. Further investigation into the underlying mechanisms and consequences of this phenomenon in an in vivo model is needed to fully understand the immunotoxic effects of Si/SiO_2_ QDs. 

Amongst the other cytokines involved in chemotaxis and cell recruitment, eotaxin, MCP-1, MIP-1α, MIP-1β, and KC did not demonstrate significant modulation upon QDs exposure in RAW 264.7 cells when assessed from the cell culture media. MCP-1, MIP-1α, and MIP-1β were constitutively expressed at high levels in RAW 264.7 cells (Supplementary Table S1). 

Among the anti-inflammatory cytokines in the 23-cytokine panel analyzed, IL-10 and IL-13 are most notable. Compared to other cytokine levels, those of IL-10 and IL-13 were relatively low; nonetheless, the responses to QD challenges were time- and dose-dependent. In cell lysates, IL-10 ranged from 20.4 to 59.1 pg/mL, while in cell media, the range extended from 29.6 to 94.4 pg/mL (Supplementary Table S1). Conversely, the highest levels of IL-3 were registered in the cell media; however, fold change increases were most notable in 6 h QD-exposed cell lysates (Figure 4).

### 3.3. Modulation of Antioxidative Enzyme Activity in RAW 264.7 Cells Exposed to Si/SiO_2_ QDs

**Figure 5:**
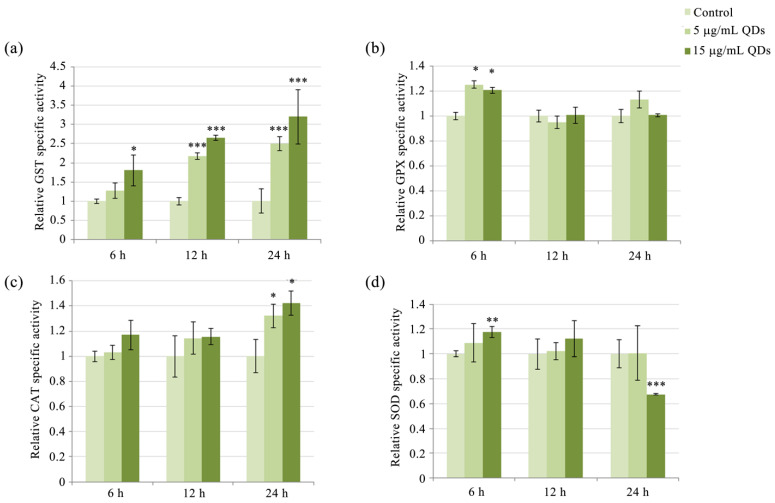
Modulation of antioxidative enzymes in RAW 264.7 cells exposed to Si/SiO_2_ QDs. (**a**) Relative GST activity levels; (**b**) relative GPX levels; (**c**) relative CAT levels; (**d**) relative SOD levels. Data are relative to control and represent means ± SD. Data represent means ± SD. Statistically significant differences (student’s *t*-test) relative to control are indicated by * *p* ≤ 0.05; ** *p* ≤ 0.01; *** *p* ≤ 0.001.

Exposure of RAW 264.7 cells to QDs resulted in a time- and concentration-dependent increase in GST activity. At 5 μg/mL, GST activity increased in a time-dependent manner and was significantly increased by 2.17- and 2.5-fold after 12 h and 24 h, respectively, whereas at 15 μg/mL, significant activity increases were noted at all time points, by 1.81-fold, 2.65-fold, and 3.2-fold at 6 h, 12 h, and 24 h, respectively (Figure 5a). The catalytic activity of GST is particularly important in macrophages, where it protects against reactive products of lipid peroxidation. Three alpha-class GST isoenzymes (the most potent against lipid radicals) have been identified, which are expressed in cell types most exposed to oxidative stress, especially macrophages, and are particularly effective against lipid hydroperoxides and 4-HNE [66]. MDA can also be generated during the catabolism of arachidonic acid by cyclooxygenase, in the synthesis of prostaglandins [67], from prostaglandin endoperoxides (PGG2) by thromboxane synthase [68], and by the breakdown of PGH2 [69]. The delayed increase in MDA levels at a QDs dose of 15 μg/mL, starting from the 12-h interval (Figure 2c), can be explained by the increase in GST activity at 6 h, which facilitates the detoxification of MDA. Furthermore, at the subsequent time points, it is plausible that the augmented levels of ROS and NO (Figure 2b,d) could have synergistically impaired the detoxifying activity of GST, leading to the accumulation of MDA and increased membrane permeability as indicated by elevated extracellular LDH activity (Figure 2a).

Another enzyme directly involved in the antioxidant defense is GPX (Figure 5b). This enzyme is more efficient than CAT in detoxifying hydrogen peroxide at low concentrations [70]. Therefore, it would be reasonable to consider that the activities of these enzymes are complementary, with GPX being activated in the case of moderate increases in cytosolic H_2_O_2_ levels, while CAT, mainly located in peroxisomes, is catalyzing the reaction of decomposition of H_2_O_2_ in the case of more pronounced increases in hydrogen peroxide levels. We observed such a complementary pattern in the activity of GPX and CAT in RAW 264.7 cells exposed to QDs. Specifically, GPX activity was activated at the early time point of 6 h (Figure 5b) and increased by 1.2-fold at both QD doses we used, while the maximum activity of CAT was recorded at the 24-h exposure interval, being 1.3-fold and 1.4-fold higher, respectively, compared to the control (Figure 5c). 

In Si/SiO_2_ QDs-exposed RAW 264.7 cells, the SOD activity was only marginally increased, by 1.18-fold, following exposure to the 15 μg/mL QDs dose at the 6-h interval, but significantly inhibited, by 0.67-fold compared to control cells, at the 24-h interval (Figure 5d). The fact that the activity of superoxide dismutase (SOD) can be negatively impacted by the presence of hydrogen peroxide at high concentrations is widely acknowledged in the scientific literature. This phenomenon is attributed to the oxidation of a histidine ligand for the copper in Cu, ZnSOD, which is a well-established and accepted mechanism [71]. It is worth noting that the inhibition of SOD activity can also be attributed to the presence of peroxynitrite, which is known to cause oxidative damage to biomolecules [72]. The fact that Cu, ZnSOD is potentially inactivated by peroxynitrite is particularly relevant in the context of our findings, as the SOD inhibition we reported may contribute to the augumentation of peroxynitrite production through the reaction between nitric oxide and superoxide. The literature has previously documented a decrease in SOD activity in RAW 264.7 cells upon exposure to CuO nanoparticles, resulting in heightened susceptibility to apoptosis induced by nitric oxide [73]. A remarkable study recently revealed that in macrophages, CuO nanoparticles induced misfolding and mitochondrial translocation of superoxide dismutase 1 (SOD1), an enzyme that plays a crucial role in oxidative stress defense [74]. Another interesting aspect revealed by the same study was that inhibition of the cellular SOD1 chaperone worsened CuO toxicity. Furthermore, the study reported that CuO nanoparticles induced the dose-dependent accumulation of polyubiquitinated proteins and reduced proteasomal function. Interestingly, CuO nanoparticles induced macrophage cell death via a non-apoptotic mechanism [74]. 

### 3.4. Key Proteins of the Stress Response Pathways Activated in RAW 264.7 Cells Exposed to Si/SiO_2_ QDs 

**Figure 6:**
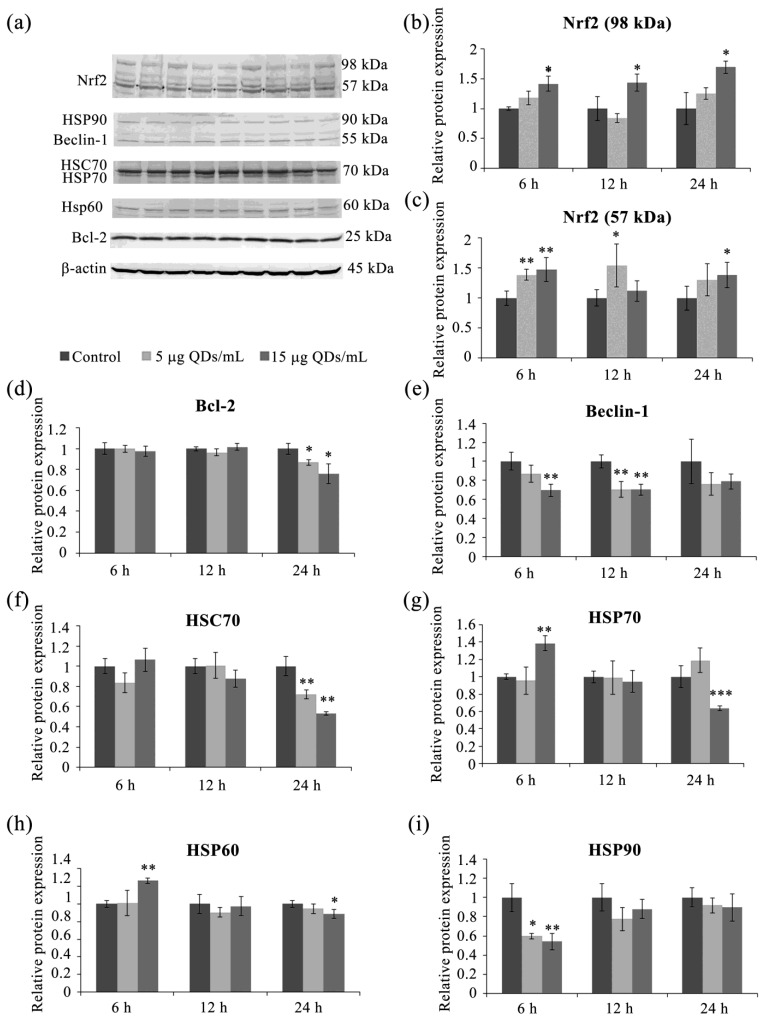
Modulation of key proteins in the stress response pathways in RAW 264.7 cells exposed to Si/SiO2 QDs. (**a**) representative immunoblots; densitometric analysis revealing the relative protein expression levels of (**b**) Nrf-2 (98 kDa); (**c**) Nrf-2 (57 kDa); (**d**) Bcl-2; (**e**) Beclin-1; (**f**) HSC70; (**g**) HSP70; (**h**) HSP60; and (**i**) HSP90. Data are relative to control and represent means ± SD. Data represent means ± SD. Statistically significant differences (student’s *t*-test) relative to control are indicated by * *p* ≤ 0.05; ** *p*≤ 0.01; *** *p* ≤ 0.001.

Cells activate protective and repair mechanisms in response to moderate stress, but cytotoxic stress can lead to apoptosis or necrosis. Nrf2 (Nuclear factor erythroid 2-related factor 2) is an essential transcription factor for oxidative stress defense, with a molecular weight of 57 kDa. Immunoblotted membranes for the Nrf2 protein indicated the presence of several immunoreactive bands, some at the expected molecular weight but also at 89 kDa (Figure 6a), which, according to the antibody manufacturers, represents polyubiquitinated forms of Nrf2 (at molecular weights of over 100 kDa). Other researchers have explored this issue and found that immunoreactive bands at high molecular weights correspond to phosphorylated forms of Nrf2 and appear after induction via oxidative stress [75]. Phosphorylation of Nrf2 is potentially involved in transcription factor activation or degradation, and the authors of that study showed that the 98 kDa form is transcriptionally active while the 118 kDa form is susceptible to degradation [75].

**Figure 7:**
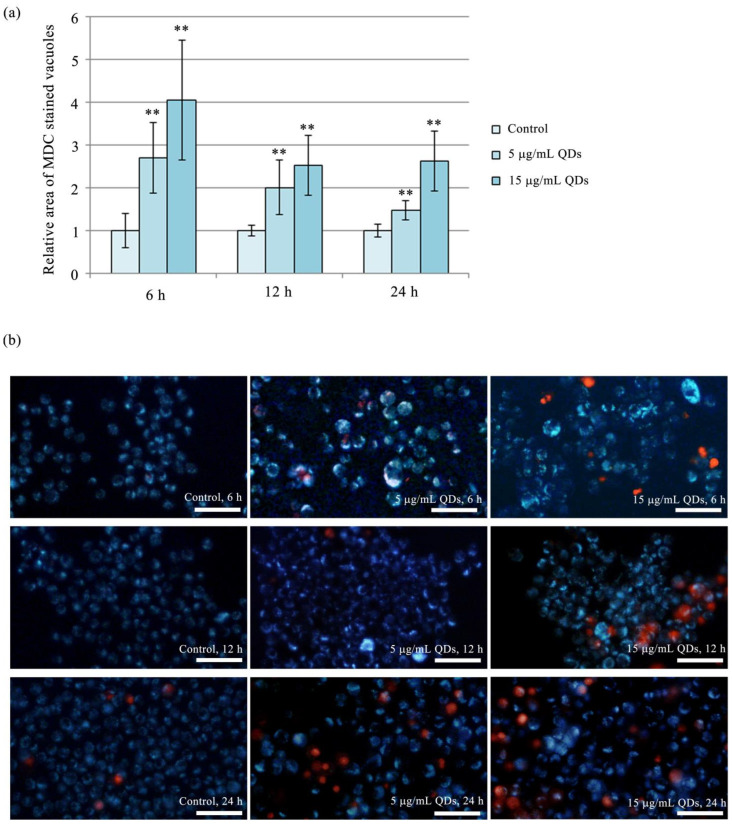
Autophagy levels in RAW 264.7 cells exposed to Si/SiO_2_ QDs. (**a**) quantification of MDC-stained vacuoles from specific fluorescence signals from fluorescence micrographs. Data represent means ± SD. A student’s *t*-test was calculated to assess statistical significance relative to controls: ** *p* ≤ 0.01. (**b**) representative fluorescence microscopy images of MDC- (in blue) and PI-stained (in red) RAW 264.7 cells exposed to Si/SiO_2_ QDs. The bar represents 50 μm.

The densitometric analysis of immunoreactive bands showed that the expression of 57 kDa Nrf2 was consistently higher than that in the control at all intervals and QD doses. After 6 h of exposure to QDs at 5 and 15 µg/mL, the expression of 57 kDa Nrf2 increased significantly by 1.39-fold and 1.47-fold, respectively. At 12 h, the expression increase was significant only at the dose of 5 µg/mL by 1.54-fold, and at the 24-h interval, only at the dose of 15 µg/mL by 1.38-fold compared to the control (Figure 6a,c). The increased expression of the 57 kDa form of Nrf2 at the early intervals of 6 h and 12 h might be due to an interaction between the Keap1/Nrf2 system and autophagy, as previously reported [76]. Specifically, the phosphorylation of the autophagy adaptor protein p62 can increase its affinity for Keap1, which complexes with Nrf2 and leads to the inactivation and proteasomal degradation of Nrf2. However, phosphorylated p62 can protect Nrf2 from binding to its inhibitor, Keap1, thus stimulating the expression of cytoprotective proteins under the control of Nrf2 [76]. Autophagy was shown to be most active at the intervals of 6 h and 12 h (Figure 7), and we can reasonably observe the similar profiles of expression of the 57 kDa form of Nrf2 and autophagy levels (Pearson’s correlation coefficient r = 0.822, *p* = 0.004). Therefore, in the context of the RAW 264.7 cells exposure to Si/SiO_2_ QDs, the Nrf2 transcription factor is activated in correlation with autophagy under moderate oxidative stress conditions at earlier time intervals and directly in response to the increase in superoxide anion levels at higher QD doses at later time intervals.

The band corresponding to the transcriptionally active 98 kDa form of Nrf2 showed a time-dependent activation at the concentration of 15 µg/mL QDs, with increases of 1.42-fold, 1.44-fold, and 1.70-fold after 6, 12, and 24 h, respectively (Figure 6a,b). Nrf-2 is a transcription factor that regulates the expression of various antioxidant and detoxification enzymes, protecting cells from oxidative stress and toxic insults. Nrf2 is a member of the Cap ‘n’ Collar family of transcription factors that regulate the expression of phase II detoxifying and antioxidant enzymes containing the antioxidant response element in the promoter region [77]. GST is one such enzyme with potent detoxifying properties, and in our study, a significant increase in its activity was observed (as shown in Figure 5a).

Although apoptosis and autophagy are often activated together in response to various stressful stimuli, the molecular mechanisms underlying their link and how they affect each other are not well understood. Several proteins have been proposed as linking molecules between these processes, including Beclin 1 and Bcl-2 [78,79], which we also focused on. The formation of the initial phagophore membrane requires the class III complex of phosphatidylinositol 3-kinase, which includes Beclin 1. Beclin 1’s involvement in autophagy is controlled by the Bcl-2 protein, which must dissociate from Beclin 1 to induce autophagy. Inhibiting Beclin 1 expression suppresses autophagy and sensitizes cells to pro-apoptotic stimuli [80,81].

In RAW 264.7 cells exposed to Si/SiO_2_ QDs, no significant changes in the expression of Beclin 1 and Bcl-2 were observed until 12 h of treatment (Figure 6a,d,e). At 24 h, the situation was different, with both proteins consistently and significantly inhibited, with Beclin 1 reaching only 0.72-fold and 0.57-fold at doses of 5 and 15 μg/mL, while Bcl-2 reached 0.87-fold of the control and 0.76-fold of the control at the same doses (Figure 6d,e). The downregulation of Beclin 1 may be a cause of autophagy decline starting at the 12-h interval (Figure 7). Bcl-2 is an anti-apoptotic protein that inhibits programmed cell death, thereby promoting cell survival. In Si/SiO_2_ QD-exposed RAW 264.7 macrophages, we observed a significant reduction in Bcl-2 protein expression levels upon 24 h exposure to 5 and 15 μg/mL (Figure 6a,d). Bcl-2 inhibition could be explained by the fact that NO is implicated in the formation of adducts with proteins belonging to the anti-apoptotic members of the Bcl-2 family, which results in diminished protein activity and culminates in their proteolytic degradation [82]. In our case, this could be a plausible explanation, as Bcl-2 inhibition was observed at the 24-h time point when NO levels were also highest (Figure 2d). This suggests that the NO deleterious effects might be reaching beyond the loss of membrane integrity, and the balance between NO production and its scavenging is critical for macrophage function and survival. 

Cellular response to stress involves, apart from changes in the expression and activity of antioxidant enzymes, the modulation of proteins involved in protein synthesis and folding. Heat shock proteins (HSPs) have been reported to be modulated in response to exposure to various nanoparticles, such as copper oxide [83], silica dioxide [84], and Si/SiO_2_ QDs [85], although in murine macrophages, another study did not report evident HSP modulation upon exposure to CuO NPs [73]. The macrophage’s specific HSP response to Si/SiO_2_ might be an important piece of the puzzle in the context of activating and executing a cellular defense or cell death program. 

We observed significant modifications in the levels of HSP60, 70, and 90 proteins in RAW 264.7 macrophages exposed to Si/SiO_2_ QDs. The expression of HSP90 was significantly decreased, reaching 0.60- and 0.53-fold of control levels, respectively, at 5 and 15 μg/mL QDs doses at the 6-h interval. Subsequently, HSP90 levels returned to control levels, and no significant changes were noted (Figure 6a,i). 

HSP60 is a mitochondrial protein induced by various stress factors, generally involved in maintaining cellular homeostasis, with an anti-apoptotic role [86]. The expression of the heat shock protein HSP60 was affected in RAW 264/7 murine macrophages exposed to Si/SiO_2_ QDs only at a dose of 15 μg/mL (Figure 6a,h). Thus, at the 6-h exposure interval, the protein expression of HSP60 increased significantly by 1.3-fold compared to the control level. After 12 h, it had similar levels to the control, while after 24 h, it was reduced, reaching 0.89-fold the control level. The role of HSP60 in inflammation is difficult to estimate, as there are studies showing both pro-inflammatory and anti-inflammatory functions [86], depending on the extracellular level of the protein. It appears that a high level of extracellular HSP60 is involved in amplifying the immune response, while reduced amounts have housekeeping and anti-inflammatory roles [86].

HSP70 and HSC70, two members of the HSP70 family, have known roles in inflammation and cell death. HSP70 has been shown to regulate the activity of the NLRP3 inflammasome, a key mediator of inflammation, and to modulate the NF-κB signaling pathway, which is essential for the expression of pro-inflammatory genes [87]. HSP70 has also been implicated in the regulation of apoptosis, acting as an anti-apoptotic factor [88]. Conversely, HSC70, a constitutively expressed chaperone, has been implicated in the regulation of autophagy [89]. In addition, HSC70 has been shown to play a role in the regulation of necroptosis [90]. 

The expression profiles of HSP70 were similar to those of HSP60, as it was only affected by the higher QDs dose, and peaked at 6 h, reaching a 1.39-fold increase compared to control, with consecutive downregulation to control levels at 12 h, and an inhibition at 24 h, when it reached a minimum of 0.64-fold of control levels (Figure 6a,g). This increase in HSP70 expression in the first interval is consistent with its chaperone and anti-apoptotic protein functions. Secondly, studies have shown that during nanoparticle internalization, HSP70 participates in stabilizing lysosomes, thereby preventing apoptosis initiation through the cellular death receptor [91]. 

The protein expression level of HSC70 has only been affected by the Si/SiO_2_ QDs after a 24 h exposure, when it was diminished to 0.72-fold and 0.53-fold of control at the 5 and 15 μg/mL QDs doses, respectively. The recognition and delivery of oxidized proteins to lysosomes are mediated by a complex of chaperone proteins, particularly HSC70/HSP70, during chaperone-mediated autophagy stimulated under conditions of moderate oxidative stress [92,93]. Considering this, it would be reasonable to conclude that the inhibition of both HSC70 and HSP70 might have contributed to the diminished autophagy levels we noted at the 24-h QD exposure interval. HSP70 has been shown to function as a SOD1 chaperone, being involved in maintaining the stability and proper folding of the enzyme and promoting its transportation to the appropriate cellular compartments [94]. Following murine macrophage exposure for 24 h to 15 μg/mL QDs, we did observe a surprising decrease in total SOD activity (Figure 5d), which could be explained by the reduced levels of both HSP70 and HSC70 in the same conditions. 

The expression of HSP90 was significantly decreased and reached 0.6- and 0.53-fold of control levels at 5 and 15 μg/mL QDs doses, respectively, at the 6-h interval, after which HSP90 levels gradually reached the control level after 24 h for both doses (Figure 6a,i). 

The role of HSP90 in inflammation and cell death is complex and multifaceted. Inhibition of HSP90 can have both anti-inflammatory and pro-apoptotic effects. The decrease in HSP90 has been shown to lead to the selective degradation of IKK by autophagy, which results in the inhibition of the NF-κB pathway, leading to an anti-inflammatory effect [95,96]. However, HSP90 is also an anti-apoptotic protein, and its decrease can induce apoptosis by inhibiting the Akt/PKB signaling pathway. Additionally, HSP90 inhibition leads to the release and activation of HSF1, which has an inhibitory effect on pro-inflammatory cytokine genes and a positive correlation with the induction of HSP70 expression [96,97]. 

### 3.5. Autophagy in RAW 264.7 Cells Exposed to Si/SiO_2_ QDs 

Autophagy is a critical cellular process that involves the degradation and digestion of damaged organelles, misfolded proteins, and dead microorganisms and plays a crucial role in safeguarding cellular homeostasis and adapting to external stressors [98]. The role of autophagy depends on its amplitude, as it is both an independent mode of programmed cell death, a protective mechanism, and a stress response activated in damaged cells [99]. A variety of inorganic nanoparticles, including Si NPs, were shown to induce autophagy in various cell types, such as HUVEC cells [100,101], L-02 cells [102], and MRC-5 [103], or disrupt it in others, such as HepG2 [104] and in vivo murine models [105], where autophagy inhibition leads to exacerbated inflammation. In addition to this, there is evidence suggesting that the inhibition of certain autophagy and apoptosis regulatory proteins can shift the balance towards necrosis in terms of the mechanism of cell death [106,107,108]. 

Considering Si-based NP have been reported to have divergent effects regarding induction or disruption of autophagy, it was important to investigate the effect of Si/SiO_2_ QDs on this critical process in macrophages. MDC-stained vacuole areas were quantified, normalized to the total number of cells, and shown relative to control cells (Figure 7a). A more pronounced presence of autophagic vacuoles was observed at the 6-h interval, with significant increases of over 2.5-fold and 4-fold upon exposure to the 5 μg/mL QDs and 15 μg/mL QDs, respectively. At the following exposure intervals, swollen cells strongly marked with MDC demonstrated that the autophagy process was still active, although at diminished levels compared to the first interval (Figure 7). 

The relative autophagy levels were negatively correlated with MDA (r = −0.921, *p* = 0.001), NO (r = −0.712, *p* = 0.015), and ROS (r = −0.835, *p* = 0.004) oxidative stress markers, as well as extracellular LDH activity (r = −0.664, *p* = 0.024). Autophagy had a moderately positive correlation with cell viability (r = 0.572, *p* = 0.049), and after peaking at the 6 h exposure to Si/SiO_2_ QDs, both demonstrated a time-dependent declining trend. 

The downregulation of autophagy levels after the 6-h interval could play a role in the loss of lysosomal integrity, which is a potential mechanism for ROS generation [109]. A previous study showed that lysosomes damaged by SiNP exposure were selectively sequestered into autophagosomes, where they could maintain their acidic pH and degradative properties. Inhibition of autophagy was correlated in that respective study with the inhibition of lysosome biogenesis in vitro and the progression of acute renal injuries in vivo [110]. Lysosome degradation has been linked to impaired autophagy in SiNP-exposed L-02 and HepG2 cell models [104].

Autophagy is functionally linked with the inflammatory process, and it was shown to play a role in the regulation of cytokine release and inflammasome activation in macrophages [111]. Based on our findings, it appears that the initiation of autophagy could potentially serve as a safeguard for RAW 264.7 cells against the detrimental consequences brought about by exposure to Si/SiO_2_ QDs, particularly by mitigating inflammation, especially in the initial stages. However, after the 24-h exposure, autophagy activity dampened, leading to the accumulation of harmful outcomes, such as oxidative damage to cellular membranes and maladaptive inflammation, ultimately culminating in cell demise.

## 4. Conclusions

The results of this study underscore the remarkable sensitivity of RAW 264.7 macrophages to Si/SiO_2_ QDs. Exposure to QDs resulted in the dysregulation of antioxidant enzymes, leading to increased levels of MDA and superoxide anion, which are indicative of oxidative stress. Concurrently, a significant loss of cellular membrane integrity was observed, as evidenced by elevated extracellular LDH activity. 

Interestingly, QD exposure also induced an increase in autophagy levels. Protein expression patterns of Beclin 1 and Bcl-2 support their involvement in autophagy regulation and cellular response to oxidative stress; their decreased expression at 24 h dampened autophagy and potentially increased macrophages vulnerability to death. HSPs also play a role in inflammation and cell death signaling, notably through diminished HSP70 and HSC70 expression.

The remarkable upregulation and release of pro-inflammatory TNF-α, G-CSF, MCP-1, and MIP-1β cytokines involved in immune cell recruitment and activation by the QDs-challenged RAW 264.7 cells suggests the potential for heightened immune activation and the possibility of a cytokine storm-like response upon exposure to Si/SiO_2_ QDs in an in vivo setting. Therefore, caution must be exercised when translating these findings to in vivo models, taking into account the dynamic and intricate immune responses that may arise. 

These considerations emphasize the need for meticulous evaluation and comprehensive assessment of the immune response when considering the application of Si/SiO_2_ QDs, particularly in the context of nanomedicine. 

## Figures and Tables

**Table 1:** Overview of methods.

Assay Domain	Assay Name	Assay Type
Cellular proliferation and viability	MTT	Colorimetric
	LIVE/DEAD	Fluorescence microscopy
	Dye exclusion	Microscopy
Stress and membrane integrity	Lactate dehydrogenase activity	Colorimetric
	Anion superoxide	Colorimetric
	Malondialdehyde	Fluorimetric
Antioxidative response	Glutathione-S-transferase activity	Kinetic, spectrofotometric
	Glutatione peroxidase activity	Kinetic, spectrofotometric
	Superoxide dismutase activity	Kinetic, spectrofotometric
	Catalase activity	Kinetic, spectrofotometric
Inflammatory response/Inflammatory markers	Cytokines	Conjugated magnetic bead-based immunoassay
	Cyclooxigenase activity	Colorimetric
	Prostaglandin E2	Enzyme-linked immunoassay
Key proteins of the stress response pathways	Nuclear factor erythroid 2-related factor 2	Western blot
	Beclin 1	Western blot
	B-cell lymphoma 2	Western blot
	Heat shock proteins 60, 70, and 90	Western blot
	Caspase 1 activity	Colorimetric
Cellular homeostasis	Autophagy	Fluorescence microscopy

## Data Availability

The data presented in this study are available within the article, Supplementary Table S1, and Supplementary Figure S1.

## Supplementary Materials

The following supporting information can be downloaded at: https://www.mdpi.com/article/10.3390/ma16145083/s1. Figure S1: Morphological changes of macrophages treated with QDs; Table S1: Multiplex cytokine assay results—absolute values, fold changes, and statistical significance.
