# Supplementary material for: Interplay of Oxidative Stress, Inflammation, and Autophagy in RAW 264.7 Murine Macrophage Cell Line Challenged with Si/SiO2 Quantum Dots
Source: Materials (Basel). 2023 Jul 19;16(14):5083. doi: 10.3390/ma16145083 (PMC10385521; doi:10.3390/ma16145083)
Supplement: Supplementary file 1 [file materials-16-05083-s001.zip › Figure S1.pdf]

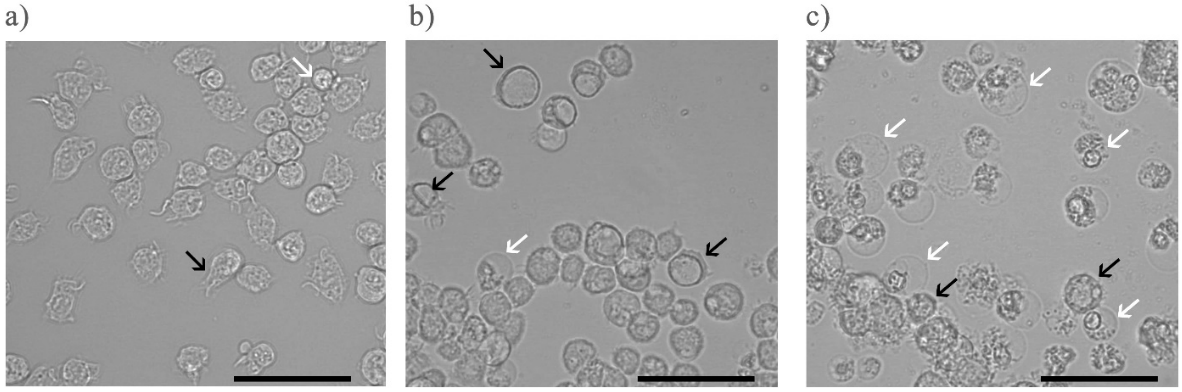

Figure S1. Morphological changes of macrophages treated with QDs. a) Control cells, at the 24 h time-point show typical morphology, with cells actively emitting filopodia (black arrow), an area featuring cellular debris (indicated by a white arrow) has elicited the migration and subsequent phagocytosis of neighboring cells. b) cells exposed to 5 µg/mL QDs for 24 h frequently show extensive vacuolation (black arows) however, the cells were still viable and continued to emit fillipodia. An example of a dead cell is indicated by a white arrow. c) QDs-treated cells at 15 µg/mL concentration for 24 h show the prevalence of necrotic cells (white arrows), while the remaining viable cells (black arrows) are rounded and lack pseudopods. The majority of cells in this field are dead. The scale bars represent 50 µm.
